# Peptides Used for Heavy Metal Remediation: A Promising Approach

**DOI:** 10.3390/ijms25126717

**Published:** 2024-06-18

**Authors:** Yingyong Luo, Yunfeng Zhang, Zhuang Xiong, Xiaodie Chen, Ajia Sha, Wenqi Xiao, Lianxin Peng, Liang Zou, Jialiang Han, Qiang Li

**Affiliations:** Key Laboratory of Coarse Cereal Processing, Ministry of Agriculture and Rural Affairs, Sichuan Engineering & Technology Research Center of Coarse Cereal Industrialization, School of Food and Biological Engineering, Chengdu University, Chengdu 610000, China; lyy1478963@126.com (Y.L.); zhangyunfeng@cdu.edu.cn (Y.Z.); xiongzhuang2000@126.com (Z.X.); cxd0512@126.com (X.C.); shaajia19980108@126.com (A.S.); xwq990713@126.com (W.X.); penglianxin@cdu.edu.cn (L.P.); zouliang@cdu.edu.cn (L.Z.)

**Keywords:** peptide repairing agent, heavy metal pollution, repair, mechanism

## Abstract

In recent years, heavy metal pollution has become increasingly prominent, severely damaging ecosystems and biodiversity, and posing a serious threat to human health. However, the results of current methods for heavy metal restoration are not satisfactory, so it is urgent to find a new and effective method. Peptides are the units that make up proteins, with small molecular weights and strong biological activities. They can effectively repair proteins by forming complexes, reducing heavy metal ions, activating the plant’s antioxidant defense system, and promoting the growth and metabolism of microorganisms. Peptides show great potential for the remediation of heavy metal contamination due to their special structure and properties. This paper reviews the research progress in recent years on the use of peptides to remediate heavy metal pollution, describes the mechanisms and applications of remediation, and provides references for the remediation of heavy metal pollution.

## 1. Introduction

Heavy metal pollution refers to the excessive presence of heavy metal elements with a density greater than 5 g/cm^3^, such as lead, mercury, cadmium, and chromium, in soils, water, and the atmosphere [1]. These heavy metals all exist in nature, but due to mining, smelting, industrial production and use, weathering, volcanic eruptions, etc. [2,3,4,5], they are released into the environment. Heavy metals are very harmful to human health; heavy metal poisoning can cause neurological diseases, cancers, malformations and mutations [6], insomnia, liver diseases, children’s mental disabilities, decreased immunity, skin diseases, depression, and visual disorders [7], and can even cause death in severe cases [8]. Heavy metal pollution not only disrupts the balance of ecosystems, harms flora and fauna, and leads to a reduction in biodiversity, but also poses a serious safety threat to agricultural soils and crops. Therefore, effective measures must be adopted to reduce the effects of heavy metal pollution.

### 1.1. Remediation Methods for Heavy Metal Pollution

There are various remediation methods for heavy metal pollution, including physical remediation, chemical remediation, bioremediation, and combined remediation. However, each of these current methods has its own limitations, and there is a need to find new solutions. In recent years, it has been found that peptides, due to their unique structure and properties, can remediate heavy metal pollution by forming complexes with metals, chelating, or reducing heavy metals. This shows great potential in the remediation of heavy metal pollution.

#### 1.1.1. Physical Rehabilitation

The physical remediation methods are mainly thermal desorption and soil replacement. Thermal desorption is a method of separating pollutants from soil by volatilizing pollutants with relatively low boiling points through direct or indirect heating from the soil [9]. This method has the advantages of a short treatment cycle, high efficiency, high safety, no secondary pollution, and recycling of the soil and pollutants [9]. Soil replacement is a short-distance replacement method for moderate quantities of extremely contaminated shallow soil, replacing the contaminated soil with uncontaminated soil to reduce the heavy metal content in the soil [10]. 

#### 1.1.2. Chemical Remediation

Chemical remediation is a method of reducing the mobility, availability, and toxicity of heavy metals in soil by injecting chemical remediation agents and utilizing the functions of adsorption, precipitation, and oxidation. Studies have shown that chemical remediation mainly includes chemical leaching, chemical stabilization, electrokinetic remediation, osmosis reaction barriers, and chemical oxidation/reduction methods. Chemical leaching has received widespread attention due to its efficiency, low cost, ease of operation, and thoroughness of treatment [11]. Injecting an extraction solution containing chemical reagents (acids, bases, salts, chelating agents, or surfactants) to dissolve or extract heavy metals from the soil is known as chemical leaching [12]. The efficiency of chemical leaching is determined by a variety of factors, including soil texture, pH, organic matter content, the presence of heavy metals in their respective forms, the type of leaching agent, leaching concentration, leaching time, and solid–liquid ratio [13]. Stabilization is the process of converting heavy metals into insoluble, immobile, and less toxic forms [14]. Stabilization has proven to be a low-cost, effective, and convenient remediation technique [15]. However, the efficiency of stability, cost-effectiveness, and adverse effects on soil need to be considered before practical application [16]. Therefore, its successful application depends largely on the choice of a stabilizer. Stabilization of heavy metals in soil is generally achieved by injecting organic stabilizers, inorganic stabilizers and organic–inorganic composite stabilizers into the soil [17,18,19,20]. Kinetic remediation-permeable reactive barriers, as an in situ soil remediation technology, have been widely used in recent years [21]. A pair of electrodes is inserted into the contaminated soil, and heavy metal ions are transferred to the electrodes by a low DC current (or pulsed electric field) or a low DC voltage gradient, known as EKR [22,23]. Heavy metals such as lead, cadmium, chromium, zinc, copper, and mercury can be effectively removed from soil by this technology [24]. The environmental availability, toxicity, and mobility of heavy metals are reduced through chemical oxidation or reduction. Heavy metals, especially redox-sensitive metals or metalloids, can be effectively removed [25]. The main factors determining the oxidation/reduction process are the physicochemical properties of the soil, the characteristics of the heavy metals, the concentrations of the heavy metals and oxidizing/reducing agents, pH and temperature. In general, dissolved oxidation dominates in acidic environments, while adsorptive and catalytic oxidation dominate in alkaline environments [25].

#### 1.1.3. Bioremediation

Bioremediation is the conversion of pollutants into harmless substances or the reduction of their toxicity and concentration through the metabolic activity of organisms or the interaction between organisms and pollutants, thereby achieving the purpose of environmental restoration. Commonly used bioremediation methods include microbial remediation, phytoremediation, and animal remediation, e.g., Leong and Chang (2014) [26] reviewed the latest progress and research on the remediation of heavy metal contamination by microalgae, described the mechanism of microalgae remediation of heavy metal pollution, and discussed the tolerance and response of different microalgal strains to heavy metals and their bioaccumulation abilities, providing suggestions for the development of efficient and commercially feasible techniques for heavy metal bioremediation. Yashikaa et al. [27] reviewed the research progress of phytoremediation of heavy metal pollution and mainly investigated the tolerance of plants to higher concentrations of heavy metals and their response to heavy metal accumulation, as well as the different mechanisms of plant tolerance to heavy metals. Li et al. [28] studied the availability, bioavailability, and leachability of heavy metal pollution in soil through the use of pig manure and earthworms. Compared with traditional soil remediation methods, bioremediation has the advantages of low cost, high efficiency, and strong sustainability. However, bioremediation also has its deficiencies, which are listed in Table 1.

#### 1.1.4. Combined Restoration

Common remediation methods adopt a single remediation method. Although the remediation method can achieve certain results, the remediation effect is not optimal, the price is expensive, and the soil properties may be changed [29,30,31]. Combined remediation is a technique that combines various remediation techniques, such as physical, chemical, and biological remediation methods, to address a specific remediation task or problem. This improves the overall effectiveness and efficiency of the remediation process. This technique is particularly useful in complex restoration projects where multiple factors and variables need to be considered and addressed in a coordinated manner [10]. Common combinatorial restoration methods include plant combinatorial restoration, electrical energy combinatorial restoration, and nano-combinatorial restoration. 

##### Plant Combinatorial Restoration

Phytoremediation includes plant-nanomaterial combined remediation technology, plant-microbe combined remediation technology, and plant-electric combined remediation technology. Currently, joint plant-nanomaterial remediation is mainly studied for lead and cadmium pollution. Zhu et al. [32] reviewed the combination of plants and nano-materials to remediate heavy metal pollution in soils, and the effect of nano-materials on plants was investigated. Joint microbial-plant remediation can improve the tolerance of plants to pollutants and promote the absorption of nutrients by the plant root system, thus increasing biomass. In addition, microorganisms can transfer pollutants from the soil to the above-ground part and enrich, transform, and utilize them in plants, effectively completing environmental pollution remediation [33]. 

##### Electrical Energy Combinatorial Restoration

Combined electrokinetic remediation includes electrokinetic-elution combined remediation, electrokinetic-joint remediation with a strong osmotic response, electrokinetic-plant joint remediation, and point electrokinetic technology combined with microbial or phytoremediation. Among these four technologies, electrokinetic-microbial joint remediation is mostly used for the removal of organic pollutants, oil pollution, and heavy metal ions such as Pb and Cd. Electrokinetic-microbial joint remediation can improve the remediation effect and economic benefits of heavy metal-contaminated soils and can also overcome the problems of long phytoremediation times and limitations of remediation effect by root depths [34,35]. For example, bacteria and humus were used as natural enhancers, and the Cr(VI) treatment efficiency increased to 90.67% after 8 days of inoculation with the reducing bacteria Microbacter sp.Y2 [36].

##### Nano-Combinatorial Restoration

The combination of nano-remediation and other remediation technologies is a promising and sustainable soil remediation technology. Nano-materials have a high adsorption capacity and reactivity, and can quickly remove environmental species from soil and water through adsorption, co-precipitation, and redox reactions. They can also remove a variety of organic and heavy metal pollutants [37,38]. Nano-scale zero-valent iron (nZVI) is one of the most studied materials for environmental purification [39,40]. It has a small particle size, a large specific surface area, strong adsorption and reduction abilities, high activity, and high pollution reduction ability [41]. Sun et al. [42] investigated the use of biochar and nano-scale zero-valent iron for the removal of the heavy metal chromium from water and soil. This included preparation and experimental factors, optimization methods, chromatographic column studies, and the removal mechanism of chromium (VI). The results showed that biochar could stabilize nano-scale zero-valent iron and enhance its electron transfer, thus effectively removing Cr(VI). Burnt et al. [42] studied the enhancement of soil erosion by nZVI soil pretreatment for soil remediation. The results showed that the addition of nZVI improved soil erosion efficiency and the weight recovery rate from 8% to 55% when the particle size fraction was between 500 and 2000 μm. 

In general, combined restoration is more promising than physical, chemical, and biological remediation, but it has its own advantages and disadvantages. Table 2 lists the advantages and disadvantages of physical, chemical, biological, and combined remediation. At present, studies on joint remediation are limited to laboratory studies, and there are few studies on field experiments. Therefore, we need to explore other new and effective methods of heavy metal remediation, rather than being limited to the joint remediation. In recent years, studies have found that some specific peptides can coordinate with heavy metal ions to form stable complexes, thereby reducing their toxicity and activity and alleviating heavy metal pollution; studies have also found that some peptides have a very strong adsorption capacity and can effectively adsorb heavy metal ions and remove them from the environment. In addition, some specific peptides can also activate the antioxidant defense system of plants and enhance their resistance to heavy metals. In summary, the use of peptides to remediate heavy metal pollution has great potential and provides new research directions and ideas for the remediation of heavy metal pollution.

### 1.2. Peptide

**Figure 1:**
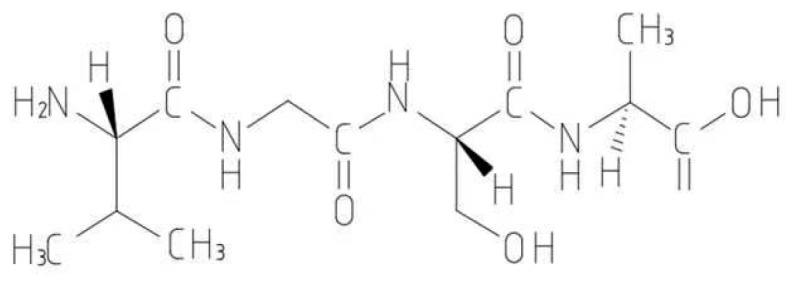
Schematic diagram of peptide structure.

Proteins are one of the most important organic substances in cell structure. It is the scaffold and the main substance that constitutes the tissues and organs of the human body and plays an important role in human life activities [43]. Amino acids are the basic units of proteins. In the study of proteins, intermediate substances different from proteins have been discovered. These biochemical substances, which are between amino acids and proteins, are called peptides. Their molecular weights are smaller than those of proteins and larger than those of amino acids. Their molecular weights range from 180 to 5000 Da, and they are biologically active components of the protein structure. The structure of a peptide consists of an amino group (NH2) and a carboxyl group (COOH) condensed by a peptide bond (Figure 1). Peptides have a variety of functions, including aiding in human growth and development [44], regulating the emotional state of stress [45], and stimulating an immune response [46]. They are also involved in regulating of cellular processes such as neuronal signaling, immune response, and growth [47]. Peptides show potential in repairing heavy metal pollution due to their structures. For example, the N-terminal amino group, C-terminal carboxyl group, amino-acid side chains, carbonyl groups, and imino groups in the peptide chain can react with the metal and remove heavy metals through the formation of complexes, chelation, or reduction, thus facilitating the remediation of heavy metal pollution. This paper reviewed the research progress and some applications of peptides in repairing heavy metal pollution and found several peptides that bind to heavy metal ions with high affinity. These peptide molecules can form stable coordination complexes with heavy metal ions, thereby removing them from the environment. This method can be used for controlling heavy metal pollution in water and soil. It has also been found that some peptide molecules can remove pollution by adsorbing heavy metal ions. These peptide molecules have specific structures and functions and can strongly interact with heavy metal ions, causing them to be adsorbed on the surface of the peptide molecule. This method can be used in wastewater treatment and soil remediation. Additionally, peptide synthesis is commonly used in the production of biological drugs, epitope mapping, peptide microarrays, and vaccine development. This study reviewed the adsorption mechanism of heavy metals by peptides and provided a reference for future use of peptides in removing heavy metal pollution and other pollutants.

## 2. Treatment of Heavy Metal Pollution by Peptides

Heavy metals are “carcinogenic, teratogenic, and mutagenic” pollutants that are extremely biotoxic and have toxic effects on humans and plants. Heavy metals at concentrations of 0.001 to 0.1 mol/L can cause serious harm to the body [48]. The main methods for removing heavy metals include chemical precipitation [49], ion exchange [50], reverse osmosis [51], microbiological methods, and adsorption [52,53], but the ubiquitous problems are cumbersome operation steps, expensive raw materials and equipment, a propensity to cause secondary pollution, and an inappropriateness for low-concentration ions. Therefore, it is urgent to find a safe, convenient, and environmentally friendly method for removing heavy metals. Amino-acid polymers and peptide materials have received widespread attention due to their advantages, such as a wide range of sources, environmental protection, low cost, and simple operation. 

### 2.1. Amino Acids Can Remove Heavy Metals

In order to remedy the issues with existing heavy metal removal methods, researchers and scholars have begun to focus on economical and practical adsorbent materials, such as activated carbon [54], cellulose [55], humic acid resin, and maitake stone. These materials form coordination or complexation with heavy metal ions through functional groups such as -OH, -COOH, and -SH on their surfaces, allowing for effective adsorption and removal. Unfortunately, the high cost of activated carbon and the need for functional group grafting of cellulose, among others, have limited the widespread application of this method. Amino-acid polymer materials are widely available, natural, and environmentally friendly, with the advantage of removing heavy metals from water without the need for modification or complex operations [56,57]. The acquisition channels mainly come from natural acquisition and artificial synthesis [58]. Natural amino-acid polymers are mainly extracted from animal tissues, including proteins, peptide hormones, enzymes, and active peptides. Synthetic amino-acid polymers are polymerized by combining multiple amino acids under specific conditions to obtain polymer compounds with multiple hydroxyl, amino, and carboxyl groups.

### 2.2. Peptide Removal of Heavy Metals

Metal-binding peptides come from a wide range of sources, including metal-binding sulfur proteins (MTs) [59], metallothionein-like proteins, phytocomplexins (PCs), and metal resistance regulatory proteins, which are all effective adsorbents of heavy metals. Many researchers and scholars have demonstrated that peptides and metal-binding proteins have better adsorption of heavy metals. Metal-binding peptides, which can be natural peptides obtained from resistant strains or artificially modified synthetic peptides, have been shown to have higher efficiency. Using bioinformatics technology and methods, such as sequence information comparison, the known metal-binding peptides were analyzed, and the results showed that these peptides were often rich in histidine. In addition, the composition of the peptides mainly included histidine, glutamic acid, proline, and alanine. This provides a theoretical basis for the interaction between metal-binding peptides and heavy metal ions. Glutathione is a tripeptide consisting of glutamic acid, cysteine, and glycine [60]. Existing studies have confirmed that glutathione has multiple atoms that can participate in coordination, and it can also produce electrochemical effects while forming complexes with metal ions. Phytochelating peptides, a phytoremediation technique, can relieve heavy metal pollution [61] and have become a hot topic of environmental governance research worldwide. In summary, peptides have great potential in remedying heavy metal pollution.

## 3. Sources of Peptides

Peptides are important structural units of proteins in organisms and have various physiological functions. According to the literature estimates, nearly 7000 naturally occurring peptides have been discovered [62,63,64], including hormones, neurotransmitters, anti-infective agents, and growth factors. These peptides play an important role in the growth of plants and animals. The sources of polypeptides are mainly animal and plant peptides, microbial fermentation, and chemical synthesis.

### 3.1. Animal and Plant Peptides

Animal peptides are extracted from animal tissues or body fluids, such as hair, skin, bones, internal organs, and blood, through acid–base hydrolysis or enzymatic hydrolysis. Animal protein peptides, as one of the main sources of bioactive peptides, currently have a wide range of applications in the food and cosmetic fields. In recent years, the development and application of bio-enzymatic digestion technology has promoted the basic research and industrialized application of animal protein peptides, which have gradually become a research hotspot in the health food industry. In addition, animal protein peptides also greatly utilize the by-products of meat processing, such as animal skins, bones, offal, etc., which improves the efficiency of resource utilization and reduces environmental pollution [65]. In recent years, with increasing attention to the clinical and pharmacological research of animal drugs, the study of their active ingredients has developed rapidly. Using fish and animal organs, many functional foods, health products, and pharmaceuticals have been developed, such as deep-sea fish collagen peptides [66], myocardial peptides for injection, blood peptides, and thymosin. The most studied animal protein peptides are milk peptides [67], insect peptides [68], meat peptides [69], egg peptides [70], fish peptides [71], and marine biological peptides [72]. Plant peptides are biologically active peptides prepared from plants as raw materials, and are safer than synthetic peptides. Soy peptides are the most common plant protein peptides [73], followed by corn peptides [74], buckwheat peptides [75], chickpea peptides [76], etc. Plant peptides have characteristics of high safety, rich nutrition (they contain eight kinds of amino acids that the human body cannot synthesize by itself and can be directly absorbed by the human body, regulating intestinal flora, enhancing human immunity, and having strong antioxidant, free-radical removal, anti-fatigue, and anti-aging effects), and low cost, and have been widely researched by modern food and pharmaceutical industries. There are many bioactive peptides in plant and animal peptides that have great potential for application in the remediation of heavy metals.

### 3.2. Microbial Fermentation

The history of microbial fermentation of peptides can be traced back to the early twentieth century. In the early 20th century, scientists began to study methods to use microorganisms, such as yeast, bacteria, and fungi, to produce natural peptides through fermentation. In the 1930s and 1940s, scientists discovered a series of important antibiotics, such as penicillin [77], streptomycin [78], and tetracycline [79]. These antibiotics are all produced by microbial fermentation, which provides an important basis for the study of peptide production by microbial fermentation. By the 1970s, the development of recombinant DNA technology provided a new method for the production of peptides through microbial fermentation. Scientists can insert target genes into the genomes of microorganisms to prompt them to produce specific peptides. In the 1980s, the further development of genetic engineering technology made the production of peptides by microbial fermentation a reality. Scientists can introduce target genes into common microorganisms, such as *Escherichia coli*, to achieve large-scale production of various peptides. With the continuous advancement of technology, important progress has been made in the industrial production of peptides produced by microbial fermentation. Fermentation techniques can be divided into solid-state fermentation (SSF) [80] and submerged fermentation (SmF) [81].

The microbial fermentation method is a technique that utilizes proteases produced by microbial strains to hydrolyze substrate proteins, thereby preparing bioactive peptides [82]. During the metabolic process, microorganisms are capable of generating intricate enzyme systems that release high concentrations of specific bioactive peptides through enzymatic hydrolysis, thereby enhancing the efficiency of the entire process [83]. At the same time, microorganisms can also produce peptidases to remove bitter peptides, making bioactive peptides have a better taste and flavor. During the fermentation process, the microorganisms themselves can also synthesize and secrete small peptides. In addition, the metabolites of microorganisms can also improve their biological activity through the modification of certain functional groups [84]. Studies also show that fermentation can also improve nutritional status and reduce antinutrient contents while increasing food variation [85,86,87]. At present, the commonly used microorganisms include *Brevibacillus laterosporus*, *Lactobacillus plantarum*, *Lactobacillus sake*, and *Lactobacillus campestris* [88,89,90]. Compared with other methods, the fermentation method has the advantages of a wide range of sources, uniform fermentation products, superior flavor, and low cost, making it suitable for industrial production [91].

Currently, there are fewer types of peptides used for the remediation of heavy metal pollution, and it is difficult to obtain them. However, microbial fermentation has many advantages such as high efficiency, environmental protection, stability, diversity, high purity, ease of control, and good biological activity. Therefore, we can prepare different types of peptides through microbial fermentation to remediate heavy metal pollution, for example, the preparation of ACE-inhibitory peptides. Bao et al. [92] fermented soy milk using 10 different strains of Lactobacillus casei, respectively. The fermentation products were determined to have some ACE inhibitory activity. Wang et al. [93] performed solid-state fermentation of soybean meal using *Bacillus subtilis* natto. The product was further subjected to ultrafiltration, dextran gel chromatography, and RP-HPLC separation and purification steps. The ACE inhibitory activity of the peptide was measured to be 84.1%, with an IC50 value of 0.022 mg/mL. The peptide was also found to be effective in inhibiting ACE. To prepare immunoreactive peptides, Elfahri et al. [94] used three different strains of *Lactobacillus swissii* and their crude enzyme extracts (CPE) to ferment milk proteins at 37 °C for 12 h. They found that the resulting short peptide mixtures induced an increase in the content of IFN-γ and the secretion of IL-10 in human peripheral blood mononuclear cells (PBMCs).

### 3.3. Chemical Synthesis

Heavy metals are harmful to both the environment and human health, so effective remediation methods need to be developed to reduce heavy metal pollution. Polypeptides, which are polypeptide chains composed of amino acids with the ability to specifically recognize and chelate metal ions, can be used for heavy metal remediation. Peptide synthesis is the key technology used to prepare peptide molecules with specific sequences and structures. Therefore, we can synthesize peptides with specific functions and specific peptide sequences by using synthesis technology to design materials with efficient adsorption of heavy metal ions for remediation of heavy metal pollution in water and soil. Thus, peptide synthesis technology plays an important role in the field of heavy metal remediation and provides new ideas and methods for solving heavy metal pollution problems. 

#### 3.3.1. Solid-Phase Synthesis Method

In 1963, Robert Bruce Merrifield introduced the concept and implementation of solid-phase peptide synthesis, which led to his earning the 1984 Nobel Prize in Chemistry. Over the past few decades, with continuous advancements, solid-phase synthesis has evolved into a highly efficient set of techniques for synthesizing peptides and even small molecules [62,63,64,65]. Two commonly used protecting groups in solid-phase peptide synthesis are Fmoc (9-fluorenylmethyloxycarbonyl) and Boc (tert-butyloxycarbonyl) [66]. The inclusion of these abbreviations and their respective numbers can provide valuable insight into the specific chemistry involved in these synthesis processes. Solid-phase synthesis plays an important role in the development of combinatorial and high-throughput chemistry and provides molecules for chemical biology, medicinal chemistry, and many other research areas. Solid-phase peptide synthesis is the primary source of peptide synthesis in laboratories, and it is also the most widely used method of chemical peptide synthesis. The first amino acid is immobilized on the solid-phase support, and then other amino acids are gradually added in a specific order to link them together by the formation of peptide bonds. The most commonly used methods are FmocSPSS solid-phase peptide synthesis, Boc solid-phase peptide synthesis, the linear synthesis method, and the parallel synthesis method. The Boc synthesis method uses TFA (trifluoroacetic acid)-removable Boc (tert-butoxycarbonyl) as the α-amino protecting group, and benzyl alcohols for side chain protection. In the synthesis, a Boc amino-acid derivative is covalently crosslinked to a resin, the Boc is removed by TFA, the free amino terminus is neutralized by triethylamine, and then the next amino acid is coupled by DCC activation. The final deprotection is mostly conducted by the HF method or the TFMSA (trifluoromethanesulfonic acid) method. Many biomolecules, such as active enzymes, growth factors, and artificial proteins, have been successfully synthesized using the Boc method. The difference between the Boc synthesis method and the Fmoc synthesis method is that the latter uses alkali-removable Fmoc (9-fluorenylmethoxycarbonyl) as the protecting group for the α-amino group, and tert-butoxy, which is removable with TFA, for the protection of the side chain. The resin used in this method is a 90% TFA-removable p-alkoxybenzyl alcohol type resin. The final deprotection step avoids the use of strong acids. However, FMOC SPPS is the preferred method for peptide synthesis, as it is widely used and effective [95]. The main characteristics of solid-phase synthesis include the following: (1) first, the building blocks are anchored on the matrix, which can be encapsulated therein; (2) repetitive chemical transformation cycles, especially deprotection and coupling reactions, are performed through automation; (3) the most common way is to release the final product in the matrix and deprotect it in the same step. However, through appropriate selection of protecting groups and linkers, the peptide can also be deprotected while remaining linked to the support or released from the support in a fully protected form. However, SPPS also has some disadvantages, such as too many reagents involved and a large amount of solvent needed for each synthetic step [96].

#### 3.3.2. Solution-Phase Synthesis Method

SPPS can easily be scaled up, and it allows for the multi-kilogram production of a peptide at an average of one residue per day [97,98,99]. However, SPPS is also associated with several drawbacks, including the large excess of reagents involved and the huge amounts of solvents required in each synthetic step. Therefore, new synthetic routes need to be developed. Liquid-phase peptide synthesis is emerging. The reactions of liquid-phase peptide synthesis are also carried out in solution, thus avoiding the use of excess solvents. The basic criterion for the LPPS strategy is the nature of the soluble label, which must be different from the nature of the reagents and by-products produced during the reaction process in order to facilitate their removal by simple precipitation, filtration, or extraction. The liquid-phase synthesis method now mainly adopts the BOC and Z two protection methods, and is mainly used in short peptide synthesis, such as aspartame, force peptide, oxytocin, etc. Relative to solid-phase synthesis, it has many advantages, including more choices of protection groups, lower cost, and easier scalability.

Labeling has changed dramatically in recent years, from polydisperse polyethylene glycol (PEG) to monodisperse PEG. These include PEG-based peptide synthesis, membrane-enhanced peptide synthesis, fluorine technology, ionic liquids, and more. Bayer and Mutter were the first to combine the potential advantages of CSPS and SPPS by introducing, for the first time, the LPPS concept. This concept involves the use of a soluble polymer (PEG) instead of a solid support (SPPS) to carry out reactions in solution [98,99,100,101,102]. In PEG-LPPS, some PEGs exhibit solubility in a variety of solvents, including dimethylformamide (DMF), dichloromethane (DCM), toluene, acetonitrile (ACN), and water. However, they are poorly soluble in tertiary-butyl methyl ether (MTBE), ether (DEE), hexane, and isopropanol (IPA). This solubility difference is exploited for peptide-PEG purification through precipitation or filtration, followed by washing during peptide extension. Membrane-enhanced peptide synthesis (MEPS) is a variation of the PEG-labeling-based technique in which reagents are removed by organic solvent nano-filtration (OSN). This technique was developed by Livingston and colleagues [103,104,105]. The MEPS strategy uses PEG (molecular weight 2000–8000) as a label [106,107,108], which is the only strategy that uses a single reactor for all synthesis processes. Perfluoroalkyl (fluoro) labels are simultaneously fluorophilic, lipophobic, and hydrophobic [109,110,111]. This behavior allows fluorous tags to self-assemble in the fluorous phase away from the organic and aqueous phases. Fluorine-containing tags in peptides can be broadly categorized into two groups: light and heavy chains [112,113]. Room temperature ionic liquids (RTILs) are chemical entities that have a small, negligible vapor pressure, are non-flammable, have reduced friction, high chemical and thermal stability, and excellent ionic conductivity and recyclability [114,115]. Ionic liquids (ILs), also known as “designer solvents,” can be easily fine-tuned by selecting specific cations and anions, allowing them to be separated from organic and aqueous layers [116,117,118]. This property makes ionic liquids ideal for use as reagents or solvents in green chemistry reactions [118,119,120].

## 4. Mechanism of Peptide Remediation of Heavy Metal Pollution

As a bioactive molecule, peptides have various functions and application potentials [121]. In recent years, a growing body of research has shown that peptides have a unique role in repairing heavy metal pollution. The mechanisms by which peptides repair heavy metal pollution mainly include the following aspects. Firstly, the amino-acid residues in the peptide molecule have different functional groups that can form coordination bonds with heavy metal ions [122]. This selective binding ability enables the peptides to effectively capture and remove heavy metal ions from the solution, thereby reducing their harm to the environment and ecosystems. Secondly, peptides can also reduce the toxicity of heavy metals by forming stable peptide–metal complexes [123]. These complexes can reduce the activity of heavy metal ions and prevent further damage to cells and tissues. At the same time, peptide–metal complexes can promote the precipitation and immobilization of heavy metals, thereby reducing their migration and spread in the environment. In addition, peptides also have a certain reducing ability and can reduce heavy metal ions to their less toxic forms [124]. The reduction of heavy metal toxicity through various methods, such as immersion and solvent extraction, can effectively make these metals less harmful and facilitate their absorption and elimination by organisms. These techniques are employed to transform heavy metal ions into less toxic forms or complexes, often through the formation of peptide–metal ion chelates. These chelates bind to the heavy metal ions, reducing their bioavailability and potential for causing harm. By making the heavy metals more soluble or accessible to metabolic pathways, the chelates enable organisms to more efficiently remove and detoxify these potentially toxic substances.

Therefore, the mechanisms for peptide repair of heavy metal pollution are multifaceted, including the selective binding, complex formation, and reduction. These mechanisms provide new ideas and ways for the development of efficient and sustainable heavy metal pollution remediation technologies. Future research will further explore the mechanisms of peptide remediation of heavy metal pollution and strengthen the combination with other remediation technologies to achieve better remediation effects and environmental protection benefits.

### 4.1. Peptide–Metal Ion Chelate

Peptide–metal ion chelates are metal–organic compounds that are prepared by chelating metal ions with peptides. They have a variety of biologically active peptide compounds [125]. They can improve the bioavailability of metal ions through the absorption mechanism of peptides in the body and have physiological and biochemical properties that inorganic metal ions do not have. At present, chelates made by chelating essential trace elements (e.g., Fe^3+^, Ca^2+^, Zn^2+^, etc.) with peptides have become a new type of metal ion supplement and have received more and more attention. In addition, it has been found that chelation of protein hydrolysates from different sources with certain metal ions (e.g., Fe^2+^, Cu^2+^, Zn^2+^, Ca^2+^, etc.) that have antioxidant or antimicrobial activities can enhance their antioxidant, antimicrobial, and other biological activities. Therefore, peptide-metal ion chelates have the activity of promoting the uptake of metal ions and have great potential in the remediation of heavy metal pollution.

#### 4.1.1. Preparation Method

Metal-ion-chelated peptides are organic compounds with a cyclic layout formed by linking metal ions and peptides in a specific molar ratio. The main factors affecting the preparation of peptide–metal ion chelates are temperature, time, and pH. Higher purity peptide–metal ion chelates can be obtained by improving any of these factors. Within a certain temperature range, the higher the reaction temperature, the higher the reaction rate and equilibrium coefficient of the chelation reaction. However, high temperatures can lead to the denaturation of the peptide, which results in a decrease in the chelating ability of the peptide zinc [126]. The chelation rate and physicochemical properties of chelated products vary depending on the chelation process. Therefore, optimizing the production process of metal-ion-chelated peptides to obtain chelated peptides with a high chelating rate and high activity is the most important research priority. Cai et al. [127] used response surface methodology to optimize the conditions for the chelation reaction of cod skin collagen peptides with ferrous ions, and the yield of chelated peptides was 37.31%. In addition, the preparation methods for metal-ion-chelating peptides mainly include enzymatic processes. For example, Lee and Song [128] studied porcine plasma proteins hydrolyzed with a flavored protease. The hydrolysate was filtered using YM-3 membranes, and a calcium-chelating peptide was isolated and purified from the hydrolysate by Sephadex G-15, high-performance liquid chromatography, and other methods. The peptide was then cultured in situ.

#### 4.1.2. Chelation Reaction Mechanism

Initial investigations revealed that the chelation mechanism between peptides and metal ions is that the terminal carboxyl group or amino group of peptides contains a relatively large number of nitrogen, oxygen, and sulfur atoms. These atoms can form complexes with metal cations under suitable conditions. In addition, a variety of peptides that do not contain phosphate groups can also bind to metal ions through the carboxyl groups of amino-acid residues. For example, Bao et al. [129] investigated the relationship between calcium-chelating ability and carboxyl group content in soy protein hydrolysates (SPHs). The results showed that the amount of chelated calcium increased linearly with increasing carboxyl group content, and the most likely chelation sites were the carboxyl groups of aspartic acid and glutamic acid. We can determine the chelation sites of the peptide–metal ion chelates through spectral analysis. Kahlen et al. [130] molecularly docked peptide chains consisting of 10 glutamic acid molecules to simulate the structure of Ca^2+^ chelation. The size of the molecular mass first affects the chelating activity, and to a certain extent, the chelating ability is negatively correlated with the peptide’s molecular mass. Xia et al. [131] structurally characterized antioxidant peptides from barley glutenin and found that small peptides with a molecular mass of less than 1 kDa were more chelating active. Amino-acid composition is the second factor that affects chelating activity, and it has been found that chelating activity is more likely to occur in the presence of cysteine and histidine in the peptide. For example, iron-chelating peptides contained in yeast hydrolysate were found to have large amounts of histidine, lysine, and aspartic acid [132]. The carboxyl groups on aspartic acid and glutamic acid may be the binding sites for metal ions and peptides, thus significantly affecting the chelation reaction [129,133]. It was also found that the phosphorylation sites of proteins were related to the chelation reaction. The dephosphorylation of casein significantly reduced the chelation of zinc ions by casein phosphopeptides (CPPs) [134], and CPPs were the first bioactive peptide to be found to promote calcium absorption. There are three main chelation modes between peptides and metal ions: monodentate, bidentate, and alpha. In the monodentate mode, Ca^2+^ is chelated with only one oxygen atom from the COO- group. In the bidentate mode, both oxygens from the COO- group can bind to Ca^2+^. In the alpha mode, Ca^2+^ is chelated with one oxygen from the carboxylate salt and one appropriate organic atom from other calcium-binding groups (O, N, S, etc.) [135]. For example, Lin et al. [136], in their study of Fe^2+^-chelating properties and mechanisms to improve the bioavailability of Fe^2+^-chelated peptides, found that Fe^2+^ was chelated with the carboxyl oxygen atoms of Glu–Glu residues in both monodentate and bidentate chelating modes. Zhang et al. [137] purified a calcium-bound decapeptide from Pacific cod-bone gelatin hydrolysates, in which Ca^2+^ was bound to the carboxylate O-atom of Lys-10 and the amino N-atom of the side-chain, forming calcium chelated peptides through the α-chelating mode.

### 4.2. Phytochelins

The ability of plants to accumulate large quantities of heavy metals from the soil when stressed by heavy metals provides an alternative, less costly method of absorbing heavy metals directly from the soil. Currently, at least 45 plant families with more than 400 plant species are known to be metal accumulating [138,139]. Many species belonging to the family Brassicaceae have been found to accumulate moderate amounts of various metals in their tissues [140,141,142]. Several mechanisms of heavy metal tolerance and hyperaccumulation have been proposed, mainly for cadmium (Cd) [143], selenium (Se), nickel (Ni), zinc (Zn) [144], and arsenic (As) [145] in plants grown under metal stress. However, metal detoxification in plants can be achieved through the distribution of metals to extraplasmic body tissues (e.g., trichomes and cell walls) [146] and chelation of metals by ligands, followed by vesicular segregation of metal–ligand complexes [147]. Metals can complex with extracellular ligands. For example, the mechanism of aluminum tolerance is mediated by the efflux of organic acids, such as malic and citric acid, from the roots. It is also mediated by intracellular chelation, which involves peptide ligands like metallothionein (MT) and phytochelatins (PC). MT is a cysteine-rich peptide that was originally identified as a cadmium-binding peptide in mammalian tissues. Subsequently, a number of MT genes and proteins have been identified in plants [148,149]. MT is genetically encoded, and PC is enzymatically synthesized by a family of peptides first identified in the yeast fission yeast, called Cadystins [150]. PC has also been identified as a heavy metal-binding ligand in a variety of plant species, including monocots, dicots, gymnosperms, and algae [151]. Table 3 lists the classification of phytochelatins.

PCS is comprised solely of three amino acids: glutamine (Glu), cysteine (Cys), and glycine (Gly). The Glu and Cys residues are linked by γ-carboxamide bonds, and they are structurally analogous to the tripeptide glutathione. β-Glutathione (GSH; γ-glutathione) is not synthesized by ribosomes, and its formula can be expressed as (γ-Glu-Cys)n-Gly, where n ranges from 2 to 11, but generally no more than 4 to 5 (Table 3) [149,152,153,154,155,156,157]. According to the number of Glu–Cys units, PCs can be divided into PC2, PC3, PC4, PC5, and PC6 [151,154]. In addition, a number of structural variants, such as (γ-GluCys)n-β-Ala, (γ-GluCys)n-Ser, and (γ-GluCys)n-Glu, have been found in several plant species [158]. The structures of the PC and metal complexes may be different due to different metals and complexes with different degrees of polymerization [159,160,161]. For example, all peptides could form 1:1 CD–ligand complexes, but the CD–ligand complexes of glutathione, PC2, and some PC3s could all form 1:2. CD–ligand complex is a complex consisting of a CD molecule (cluster of differentiation, i.e., a cell surface marker) and a ligand (e.g., a drug, a protein, etc.). Such complexes have an important role in biomedical research and can be used in disease diagnosis, therapy and drug discovery. In addition, PC and metal complexes are more stable and less toxic than free metal ions [152,157,162]. The extent of PC aggregation and PC accumulation in plants largely depends on the physicochemical properties of the metal ions, exposure time, and metal concentration [163,164,165,166,167,168,169,170]. The PC synthase gene was identified for the first time in higher plants and fission yeast [171,172]. It was also found that PC synthase (EC 2.3.2.15) catalyzes the synthesis of phytochelatins from substrates such as glutathione (GSH) and related thiol tripeptides in the presence of metal ions, including Cd, Cu, Zn, Ag, Hg, and Pb, in cultured cells [173]. In addition, a number of biochemical and genetic studies have confirmed that GSH (or, in some cases, related compounds) is a substrate for PC biosynthesis. In particular, genetic studies of GSH-deficient mutants of the fission yeast Corn Wine have confirmed that they are PCs. Similarly, the GSH-dependent activity of PC synthase was found in Arabidopsis [174], pea [175], and tomato [176].

Chelation of heavy metals with PCs is a major cellular mechanism for heavy metal detoxification and may act synergistically with secondary stress defense antioxidant systems against heavy metal-induced oxidative stress. It has been reported that the stress produced by low Cd accumulation in wheat leaves can be counteracted by antioxidant responses and PC biosynthesis. In contrast, excess Cd leads to a significant increase in H2O2 despite the increased presence of PCs and related thiol-peptide compounds in roots [177]. Mishra et al. observed an increase in the levels of antioxidant enzymes (i.e., superoxide dismutase, guaiacol peroxidase, ascorbate peroxidase, and glutathione reductase) and phytochelatins (PCs) in Pseudoamaranthus at initially low Cd concentrations. There was a gradual decrease in the levels of PCs as the concentration and duration of exposure and oxidation increased [178]. Therefore, it can be concluded that antioxidant systems are effective at lower metal concentrations, depending on the plant–metal system. Currently, the detoxification of heavy metals by phytochelating peptides in some plants, especially the detoxification of Cd^2+^, has been confirmed in various ways [165,179,180]. However, the present studies mostly focus on the mechanism of action of PC in the presence of a single heavy metal. In reality, a variety of heavy metals are often present at the same time. Therefore, the mechanism of action of phytochelatable peptides (PC) in plants under the situation of compound pollution should be strengthened in future studies.

**Table 3:** Classification of phytochelatins. Reproduced from [181].

PC Family	Peptide Structure	Identification
Phytochelatins PCn-Gly	(γGlu-Cys)n-Gly	PCn
Iso-phytochelatins		
PCn-Ser	(γGlu-Cys)n-Ser	iso-PCn(Ser)
PCn-Ala	(γGlu-Cys)n-Ala	iso-PCn(Ala)
PCn-βAla	(γGlu-Cys)n-βAla	iso-PCn(βAla)
PCn-Glu	(γGlu-Cys)n-Glu	iso-PCn(Glu)
PCn-Gln	(γGlu-Cys)n-Gln	iso-PCn(Gln)
PCn-Asn	(γGlu-Cys)n-Asn	iso-PCn(Asn)
PCn-Cys	(γGlu-Cys)n-Cys	iso-PCn(Cys)
des-Gly-PCn	(γGlu-Cys)n	des-Gly-PCn
des-γGlu-PCn-Gly	Cys-(γGlu-Cys)n−1-Gly	des-γGlu-iso-PCn(Gly)
des-γGlu-PCn-Ser	Cys-(γGlu-Cys)n−1-Ser	des-γGlu-iso-PCn(Ser)
des-Cys-PCn-Glu	Glu-(γGlu-Cys)n−1-Glu	des-Cys-iso-PCn(Glu)

**Figure 2:**
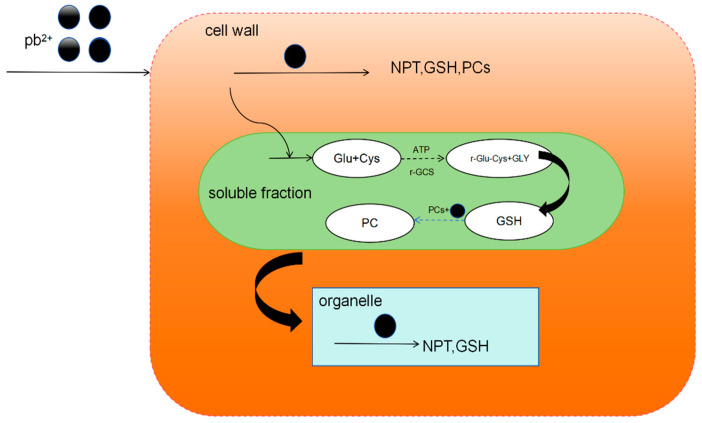
Chelation mechanism of chelating peptide from *Chladophora* plant.

The chelation of heavy metals and PC is the main cellular mechanism for heavy metal detoxification, which can synergize with the secondary stress defense and antioxidant system to counter heavy metal-induced oxidative stress. Take Cladophora plants as an example. Under lead stress, Pb^2+^ first enters the cell wall. Sulfur-containing proteins, such as NPT, GSH, and PCs, are produced in the cell wall, the main effect of which is theproduction of *NPT.* Neopterin (*NPT*) is a member of the pteridine class and is synthesized by macrophages in response to interferon gamma (INF-γ) stimulation. The *NPT* content in the cell wall rises rapidly to bind to Pb^2+^ in the cell wall, while GSH has a relatively insignificant effect at low concentrations of Pb^2+^. Phytochelated peptides will form complexes containing Pb–S and Pb–N in the cell wall. Then, it is transported by the carrier proteins in the cell membrane into the cytoplasm of the cell. In the cell fluid, PCs play a leading role. The PCs in the cell fluid will chelate the Pb^2+^ that has entered the cells to form gases containing COO–Pb, Pb–S, and CO–Pb chelates. Less Pb^2+^ entered the organelles, where Pb–S complexes were also formed. Under Pb^2+^ stress, the phytochelating peptides in Cladoides sp. chelate Pb^2+^ to form complexes containing the species Pb–S, Pb–N, COO–Pb, and CO–Pb to reduce the toxicity of intracellular Pb^2+^ (Figure 2). Naturally occurring metal hyper-enriched plants have recently become increasingly important, as they have great potential for phytoremediation of heavy metal-contaminated sites.

### 4.3. Peptide Reduction of Metals

Peptides are biomolecules that can act as reducing agents to reduce metal ions. The amino-acid residues in peptides are reduced and can form complexes with metal ions, thereby reducing the metal ions to metal atoms. Specifically, the amino functional group (-NH2) in the amino-acid residues of the peptides can form complexes with metal ions. In this complex, the oxidation state of the metal ions is reduced to the form of a metal atom. This process involves the donation of a hydrogen atom in the amino-acid residue, which reduces the metal ion to a metal atom.

The amino acids or groups in proteins that can bind to metal ions are mainly as follows. 

1. Cysteine: Its sulfur atom has an affinity and can form coordination bonds with metal ions. Especially in the protein structure, the sulfur atoms of two cysteine residues can form a disulfide bond to form a stable metal-binding site. 

2. Histidine: the nitrogen in its imidazole ring atoms can form coordination bonds with metal ions [182]. The nitrogen atom of histidine can donate a lone pair of electrons to form stable complexes with metal ions [183].

3. Glutamic acid and aspartic acid: Their carboxyl groups can form coordination bonds with metal ions. Their carboxyl oxygen atoms can donate a lone pair of electrons to form stable complexes with the metal ions [184,185].

4. Lysine and arginine: Their amino groups can form coordination bonds with metal ions. The nitrogen atoms of their amino groups can donate a lone pair of electrons to form stable complexes with the metal ions [186].

In addition to the above amino acids, there are some other amino acids or groups that can also bind metal ions, but these are relatively rare. The existence of these amino acids or groups enables proteins to participate in biological processes such as metal ion transport, catalysis, and structural stability.

## 5. Summary and Outlook

Finding efficient and environmentally friendly remediation methods has always been a hot research topic in the field of heavy metal pollution. Although current physical and chemical remediation methods can achieve certain results in certain situations, they often have limitations such as high costs and the possibility of secondary pollution. Therefore, researchers are committed to exploring new remediation technologies. Among them, peptides have shown great potential in heavy metal pollution control and have become a hot spot for researchers. MT-2 (metallothionein-2) and TAT (transcriptional activator) are two important peptides for the treatment of heavy metal pollution. MT-2 is a low molecular weight, cysteine-rich metal binding protein widely present in organisms, with a strong affinity for various heavy metal ions such as cadmium, mercury, lead, etc. MT-2 can bind with these heavy metal ions to form stable metallothionein complexes, thereby reducing the toxicity and bioavailability of these ions in the environment. In addition, MT-2 can also be decomposed and utilized by microorganisms, with good environmental compatibility. TAT is a peptide that can penetrate cell membranes and deliver other molecules. In the treatment of heavy metal pollution, TAT can bind with specific heavy metal ion ligands or adsorbents to form complexes, and then transport these complexes to the interior of cells through its ability to penetrate cell membranes. Through metabolic pathways within cells, TAT can degrade or convert heavy metal ions into non-toxic forms. This method can not only effectively remove heavy metal ions from the environment, but also reduce the toxic effects on organisms.

There have been some research reports on the specific applications of MT-2 and TAT in heavy metal pollution control. For example, some researchers use MT-2 genetic engineering technology to construct microbial strains that can efficiently express MT-2 and apply it to the remediation of heavy metal contaminated soil. The results indicate that these strains can significantly reduce the heavy metal content in soil and improve soil quality. In addition, some researchers have also conducted wastewater treatment experiments using the complex of TAT and heavy metal ion complexes and found that this method can efficiently remove heavy metal ions from wastewater and has good stability and reusability. First of all, peptides are composed of amino acids which are highly bioactive and selective, and have a variety of functions in living organisms. For example, some peptides can act as hormones or neurotransmitters to transmit signals and regulate the physiological functions of organisms. In addition, some peptides have antibacterial, anti-inflammatory, antioxidant, and other biological activities. Secondly, peptides have various structures, which can be categorized into primary and secondary structures. Primary peptide refers to the linear sequence of the peptide chain, while secondary structure refers to the folded form of the peptide chain in space. The secondary structure of a peptide may include an α-helix, a β-fold, etc. Peptides can be obtained through chemical and biosynthetic means. The methods of chemical synthesis of peptides include solid-phase synthesis and liquid-phase synthesis. Biosynthesis of peptides refers to the synthesis of peptides through enzyme systems in living organisms. In addition, peptides show great potential in the adsorption of pollutants, and in heavy metal pollution, peptides can make heavy metal pollution mitigation by cooperating with heavy metals, reduction, etc. Peptides can adsorb pesticides and microplastics to mitigate environmental pollution, so the use of peptides to remove heavy metals has great potential. Some peptides with the ability to remove heavy metals have been found, such as MT-2 and TAT, which can form stable complexes with heavy metal ions to remove them from the environment. Peptides have great research potential in the remediation of heavy metal pollution and provide new ideas and methods for researchers to solve the problem of heavy metal pollution.

However, peptide remediation of heavy metal pollution still faces some challenges. First, the peptides found so far have limited ability to remove heavy metals, and the removal ability needs to be further improved. Second, the stability and biodegradability of peptides need to be enhanced to ensure their long-term application in the environment. Therefore, we need to further study in depth the relationship between the structure and function of peptides in order to design and synthesize peptides that are more efficient in removing heavy metals and apply them in practical remediation projects for heavy-metal pollution. To study the method of peptide removal of heavy metals, we can further research the following aspects. First, computer simulations and molecular designs can be used to find more active peptide sequences. Secondly, peptides can be combined with other materials, such as nano-materials, to improve the efficiency and stability of the heavy metal removal. In addition, the biodegradation pathways of peptides can be investigated to ensure their safety in the environment. In conclusion, peptide removal of heavy metals is a promising approach, but it still needs further research and development. With the continuous progress of science and technology and the continuous efforts and innovations of researchers, it is believed that peptides in the remediation of heavy metals will bring more hope and opportunities for environmental protection and human health. It is believed that the technology of peptide removal of heavy metals will make greater breakthroughs, providing an effective solution to solve the problem of heavy metal pollution and contributing to the improvement of environmental quality and the protection of human health.

## Figures and Tables

**Table 1:** Comparison of advantages and disadvantages of bioremediation methods.

Bioremediation Methods	Advantage	Disadvantages
Microbial remediation	Efficient, environmentally friendly, low cost, sustainable, and widely applicable	Longer restoration process, unstable restoration effect, susceptible to environmental conditions, suitable for small-scale restoration, and higher restoration costs
Phytoremediation	Green, low-cost, sustainable, pollution-free, widely applicable, and biodiversity conservation	Inefficient, species-restricted, resource-intensive, difficult to control, and risk-transferring restoration effects
Animal restoration	Efficient, widely applicable, promotes biodiversity conservation, recyclable and controllable heavy metals, does not damage soil structure, and enhances soil productivity	Inability to degrade high concentrations of heavy metals, longer remediation times, and cumulative food chain effects

**Table 2:** Comparison of advantages and disadvantages of remediation methods for heavy metal pollution.

Repair Method	Advantage	Disadvantages
Physical rehabilitation	Simple operation, short time-consumption, high efficiency, reusable	High cost, small application range, incomplete restoration, easy to cause secondary pollution
Chemical remediation	Low cost, suitable for large area remediation, no damage to soil structure	Long remediation time, remediation effect is affected by catalysts, easy to pollute the environment and destroy ecosystems
Bioremediation	Green, low cost, small damage to the environment, not easy to cause secondary pollution.	Time-consuming, limited applicability, high requirements for plant vigor, growth habit, and species
Combined restoration	Beneficial, low cost, no damage to soil environment, no secondary pollution, not time-consuming	Limited to laboratory simulations, less research on field experiments, immature technology, less consideration of restoration risks

## Data Availability

All data analyzed during this study are included in this article.
